# A New and Effective Method of Treating Phthisis Pulmonalis

**Published:** 1868-08

**Authors:** 


### A NEW AND EFFECTIVE METHOD OF TREATING PHTHISIS PULMONALIS.

Dr. Carl Both, Boston, Mass., has written a monograph, in which he pronounces the curability of consumption with the greatest confidence, through *artificial calcification.* It is a practical application of the cellular pathology, announced by Virchow; and the author's theories may be given in his own words:

"As a nation consists of millions of single individuals, each holding a superior or inferior position, each dying and being replaced without injury to the whole, so is our body a commonwealth of cells, each of which has its office; each may die and become replaced by another. As a statesman watches over each individual, and tries to improve each for the benefit of the whole, so the physician should know all cells of the body, their office, and their place. He should cause their removal in case of unfitness or decay, and prevent such cells as do not fit its general structure from entering the body."

The cells composing our body live and are sustained by the food we eat, and if we cut off our food, we cut off the nourishment of the cells. By giving different food, different effects on the cells are produced. The blood requires lime for calcifying displaced or degenerated cells by depositing that substance in them.

He wishes to be understood, *that tubercles in the lungs are composed of, and originate from, blood-globules which have escaped out of the general circulation, through the bursting of an obstructed capillary vessel. That this obstruction takes place where the respiration is suppressed.* From this the conclusion is drawn *that tubercles can nowhere originate in the lungs, except in those parts where respiration has been oppressed or has ceased.*

The natural healing process consists in the calcification of the diseased parts, so that they appear as if made of chalk, though the original cells and tissues can yet be detected by the microscope. It is evident, therefore, that in such cases, the blood must have been able to furnish a considerable quantity of lime, to provide for the calcification of the decayed parts. His treatment is divided into three sections, each of which finally support the other in their effects:—

1*st.*—The *extension and cleansing of the lung by pressing air into it.*

2*d.*—The *introduction of lime into the blood* in sufficient quantity for the calcification of the tubercles; and the *purification of the blood by higher oxidation.*

3*d.*—The *determination and regulation* of a diet to suit the particular form and degree of disease.

The direct treatment of the lungs consists in pressing the air into them by natural inspiration, powerfully stimulated by certain muscular exercises which are calculated to effect this object. If, in the case of a collapsed lung and chest, the pressure of air in the lungs is increased, that organ and the thorax will necessarily be extended; and the air will pass gently and gradually into the smallest bronchi. The air vesicles and obstructed bronchi being opened, the pus and mucus contained in them will be expelled by the increased ciliary motion, by the revolving air, and by the action of the cough. At the same time the capillary circulation will be increased, diosmosis of the cells renewed, and many of them rescued from fatty degeneration and decay.

To increase the nervous action of the lung, and to produce at the same time an increased pressure, tension of the respiratory muscles must be resorted to, as a pumping force on one side, and as an irritant on the respiartory nerves by reflex irritation on the other.

To demonstrate the result, the following practice will be found of service: Let a person rest the whole of his weight on the ends of his toes and fingers in a horizontal position. He will find, on rising, that he must take larger and more forcible inspirations than were otherwise possible. By means of the forced inspiration effected in this way, air is driven into the diseased part of the lungs, and distends them in consequence.

The treatment of the blood consists, *first*, in purifying it from those substances which cause the profuse perspiration. The food is divided into two classes—respiratory and plastic. The first is that which contains no nitrogen. The second is that which does contain nitrogen, and which replaces the materials consumed by the action of the body.

The treatment of the blood consists, *second*, in the introduction of *phosphorus* and *sulphur*.

Having provided for the introduction of sulphur and phosphorus into the blood, we are, *third*, to obtain a sufficiency of *lime*, *silica*, and *magnesia*.

These materials are abundantly found in the hulls of oats, barley, wheat, and rye; but in the early stages of the treatment these cannot be readily digested. Extracts of herbs and plants, known to be rich in these three substances, such as *Triticum repens*, *Achillea Millefolia*, *Marrubium vulgare*, *Leontodon taraxacum*, *&c.*, serve as a proper substitute. The general rule for the administration of food, in every case, should be the following: to *adjust the quantity given to the amount of oxygen to be absorbed.* For *respiratory* food, make use of whey, freshly made of boiled milk, from which the caseine has been separated by adding a little cream of tartar; malt sugar, honey, fresh butter; in the spring and summer, milk, after it has become thick by the formation of lactic acid. For *plastic* food, give Liebig's extract of meat, when the digestion is very bad! Raw meat, chopped fine, given in the form of a salad, is excellent. When the digestion is good, beef, mutton, game, and fresh fish, are the best articles of food.

The bread should be made of rye meal and corn flour (not sifted too finely). Sago, cracked wheat, farina, rice, corn and oatmeal, tomatoes, and all kinds of fresh and acid fruits, may be given as the case requires it.

A detailed statement is added of the history and treatment of twenty-one patients, between the ages of nineteen and forty-seven, who have been benefited, or entirely cured; with the exception of those who had diseases of the bowels. The author believes that by following the method described, every tubercular affection of the lung can be arrested without fail—only there must not be large open caverns.

